# Tensile strength analysis of automatic periodic stimulation for continuous intraoperative neural monitoring in a piglet model

**DOI:** 10.1038/s41598-021-84988-y

**Published:** 2021-03-15

**Authors:** Tie Wang, Gianlorenzo Dionigi, Yishen Zhao, Daqi Zhang, Antonella Pino, Henning Dralle, Che-Wei Wu, Le Zhou, Hui Sun

**Affiliations:** 1 Division of Thyroid Surgery, China-Japan Union Hospital of Jilin University, Jilin Provincial Key Laboratory of Surgical Translational Medicine, Changchun City, Jilin Province China; 2 Division for Endocrine and Minimally Invasive Surgery, Department of Human Pathology in Adulthood and Childhood “G. Barresi”, University Hospital G. Martino, University of Messina, Via C. Valeria 1, 98125 Messina, Italy; 3 Section of Endocrine Surgery, Department of General Visceral and Transplantation Surgery, University of Duisburg-Essen, Essen, Germany; 4 Faculty of Medicine, College of Medicine, Kaohsiung Medical University Hospital, Kaohsiung Medical University, Kaohsiung, Taiwan; 5 Department of Otolaryngology-Head and Neck Surgery, Kaohsiung Medical University Hospital, Kaohsiung Medical University, Kaohsiung, Taiwan

**Keywords:** Action potential generation, Surgery

## Abstract

Continuous intraoperative neural monitoring (C-IONM) during thyroid surgery is a useful tool for preventing recurrent laryngeal nerve (RLN) injury. The present study aims to analyze the tensile strength tolerance of C-IONM electrodes on the vagal nerve (VN). A C-IONM wire was enclosed in a hand-held tensile testing system. The probe displacement on the VN was continuously monitored by positioning a second probe far-up/proximally in a piglet model, and an automatic periodic stimulation (APS) accessory was used. The 3-mm and 2-mm APS accessory has a mean tensile strength of 20.6 ± 10 N (range, 14.6–24.4 N) and 11.25 ± 8 N (range, 8.4–15.6 N), respectively (*P* = 0.002). There was no difference between bilateral VNs. The mean amplitude before and during electrode displacement was 1.835 ± 102 μV and 1.795 ± 169 μV, respectively (*P* = 0.45). The mean percentage of amplitude decrease on the electromyography (EMG) was 6.9 ± 2.5%, and the mean percentage of latency increase was 1.9 ± 1.5%. No significant amplitude reduction or loss of signal (LOS) was observed after > 50 probe dislocations. C-IONM probe dislocation does not cause any LOS or significant EMG alterations on the VN.

## Introduction

Continuous intraoperative neural monitoring (C-IONM) during thyroid surgery is a useful tool for preventing recurrent laryngeal nerve (RLN) injury^1^. The placement of an electrode/probe around the vagal nerve (VN) is a crucial procedure for C-IONM, and the anatomical position of the C-IONM electrode on the VN is important for electromyography (EMG) signal stability^1–3^. Generally, the dimensions of accessories in the C-IONM system is at the millimeter level^1–17^. Electrode displacement is a common situation, while the incidence varies among reported studies, which range within 5%–41%^1–17^. Previous studies have also noted that C-IONM electrode displacement may be associated with clinical outcomes^1,3^. The intraoperative electrode displacements are multifactorial, which may be affected by probe design, probe size, materials, surgical technique, and VN features^8^. In addition, electrode displacements can also be a direct iatrogenic consequence following implanting malposition, failure of fixation, insufficient experience, misoperation, inappropriate surgical approach to the carotid sheath (medial or lateral), and prolonged surgery^17^. Thyroid surgeons have acknowledged that a corrective modification of the surgical technique should be intended to simplify and minimize these surgical procedures^6,9^. Other studies have also identified two causes of electrode displacements: size of the VN, and location of the VN in the carotid sheath^9,11^. Furthermore, the modified implantation of these C-IONM probes may also reduce the incidence of displacement. Electrode repositioning affects surgical efficacy, prolongs the operation time, increases the risk of neurovascular injury, and even requires surgical correction^6^. However, there have been no systematic investigations, especially biomechanical studies, regarding the effects of electrode dislocations on the VN. The present translational study aimed to analyze the tensile strength tolerance of C-IONM electrodes on the vagal nerve (VN) in a piglet model.

## Materials and methods

### Animal model

A female piglet (body weight, 20 kg) was obtained from the Laboratory Animal Center of Jilin University (China). For the establishment of the model, the piglet was given 1.2 kg of mash daily, and unlimited water for one week. Then, the piglet was given 0.6 kg of mash on the day before the surgery, and was treated with fasting and water deprivation on the day of the surgery. The study protocol was approved by the Institutional Animal Care and Use Committee of the Laboratory Animal Center of Jilin University (China).

### Induction and maintenance of anesthesia

Physiological parameters, including electrocardiography (EKG), oximetry, end-tidal CO_2_ and airway pressure were continuously monitored. The piglet received anesthesia induction with 0.5 mg of atropine sulfate via subcutaneous injection, and 40 mg (2 mg/kg) of zoletil and 40 mg (2 mg/kg) of xylazine hydrochloride via intramuscular injection. Then, a 6-mm endotracheal tube with electromyography (EMG) electrodes (Trivantage Tube; Medtronic, Jacksonville, Florida, USA) was properly placed. After endotracheal tube fixation, the piglet was placed in the supine position with the neck extended. The depth and angle of contact between the electrode surface of the endotracheal tube and the mucosa of the vocal cord were determined by video laryngoscopy. Muscle relaxants were avoided to prevent the inhibition of EMG signals. Isoflurane (2.0–3.0%) and oxygen (2.0 L/min) were used for the maintenance of general anesthesia. The lungs were ventilated in volume-control mode at a tidal volume of 8–12 ml/kg, and the respiratory rate was set to 12–14 breaths/min.

### Monitoring equipment

Bilateral VNs were identified using a single-use, incrementing press stimulating probe with a monopolar, standard flexible tip (Product no. 8225490; Medtronic, Jacksonville, Florida, USA). The impulse duration was 100 ms, and the frequency was 4 Hz. C-IONM was performed using automatic periodic stimulation (APS; Medtronic, Jacksonville, Florida, USA). All channel leads from the EMG tube, needle electrodes and stimulating probes were connected to a NIM3.0 monitoring system (Medtronic, Jacksonville, Florida, USA). The peak-to-peak amplitudes of evoked EMG activities were directly collected on the monitor screen and recorded. The event threshold was set with a reduced response threshold to identify small responses at 50 μV, which was adjusted according to intraoperative specific circumstances.

### Surgical procedure and VN identification

An H-shape incision was made in the middle of the neck for exposure of the thyroid and surrounding important tissues^18,19^. Then, the subplatysmal flaps were raised. A lateral approach between the sternocleidomastoid and sternothyroid was adopted to access the carotid sheath, and minimize possible interferences to the APS and the surgical field. The VNs were identified and carefully exposed using the hand-held stimulation probe, according to standard procedures. A 2.0-mA stimulating intensity was used for the localization of VNs. The RLNs were not exposed.

### APS implantation

C-IONM has been performed in our institution for many years. The present experiment was performed by senior surgeons (HD, HS and GD), who have more than 10 years of experience in neural monitoring. The APS was gently placed on the VN after opening the carotid sheath. A careful 360° dissection of the VN with forceps was achieved. In order to prevent VN thermal injuries, energy-based devices were avoided. The APS accessory was wet to facilitate its sliding into the VN. The APS electrodes were gently placed on the VN, while keeping the enclosure tabs open with forceps. After the C-IONM electrodes were placed, the VN was repeatedly stimulated using an intermittent stimulating probe, which was positioned proximally and distally to the location of APS. The potential VN injury secondary to the dissection or electrode placement was assessed.

### Study protocol design

**Figure 1:**
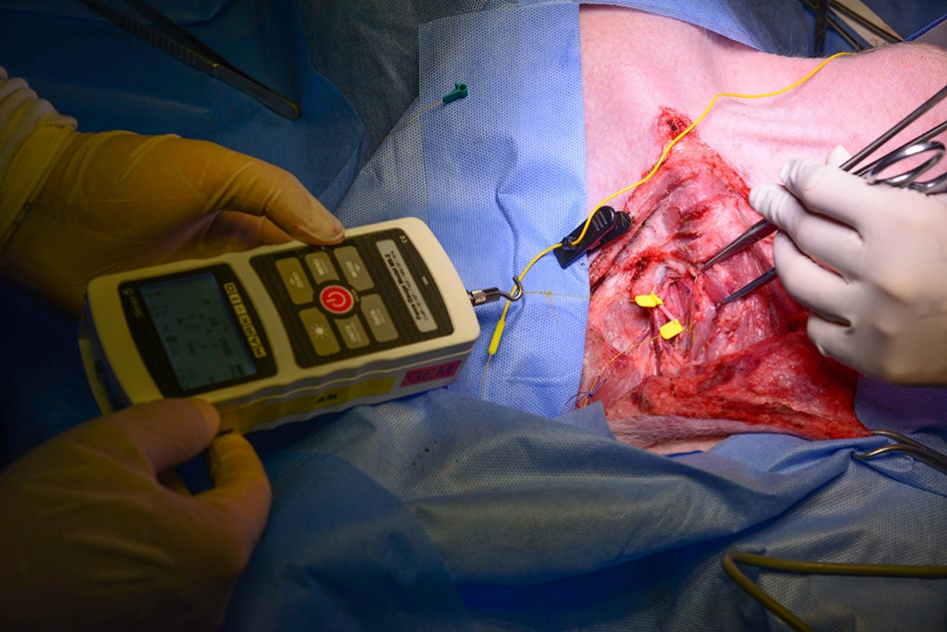
Illustration of the tensile test. The parameters and threshold levels of the VN were recorded using APS and a hand-held tensile testing system before the probe displacement test.

**Figure 2:**
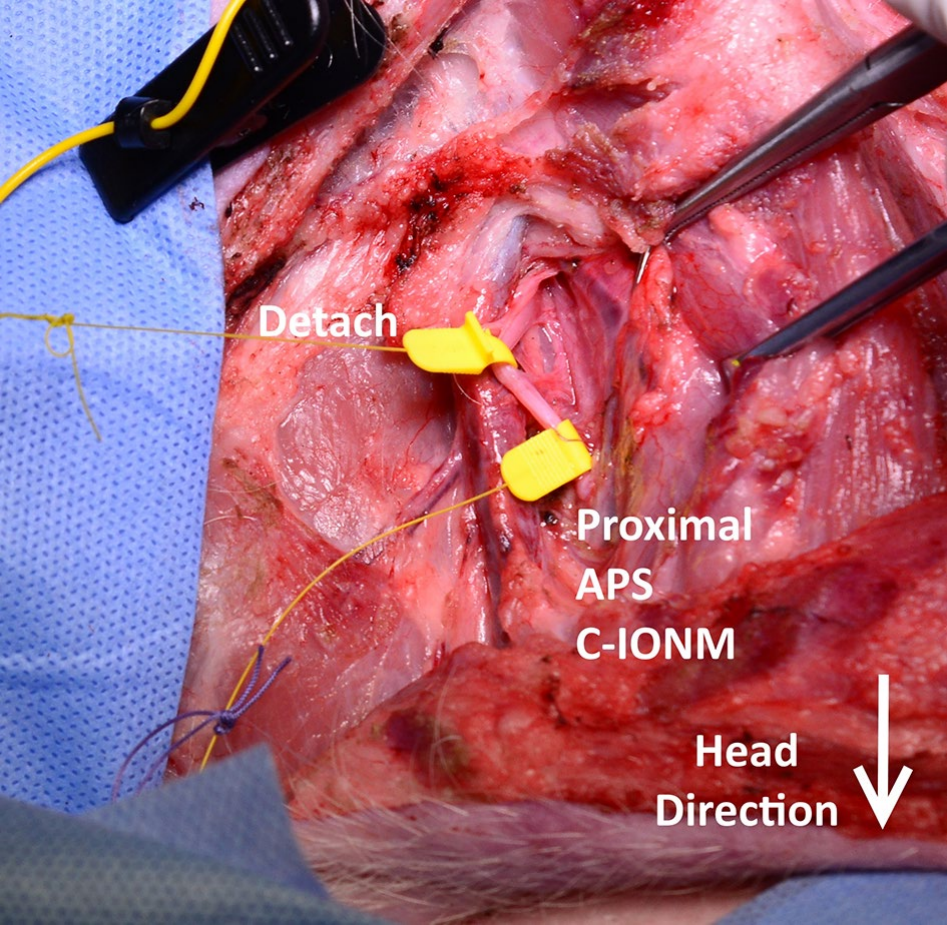
Depiction of the anatomical position of two APS, and a representative image showing the probe dislocation.

The APS wire was wrapped around a hand-held tensile testing system (ES20; Mark-10 Corporation, 11 Dixon Ave, Copiague, NY 11,726, USA), which was designed for small-size samples with 0.01-mm resolution (Fig. 1). In order to optimize the APS migration and EMG signal modification, a second APS was proximally located to observe the real-time EMG changes during traction of the distal APS (Fig. 2). The continuous monitoring of APS displacement on the VN was performed by positioning the other APS far-up/proximally to the site of the APS dislocations (Fig. 2). After connecting the second (proximal) APS electrode with the monitor system, the baseline latency and amplitude of the evoked response were recorded as control data. The stimulating frequency for C-IONM was set at twice per second, and VN integrity was constantly evaluated. In brief, merely the proximal APS periodically stimulated the vagus. The removal/displacement of the APS was performed through gradual or acute maneuvers. The protocols included multiple sequences and tests for APS dislocations (5 times and > 50 times), with 2-mm (yellow) and 3-mm (green) APS accessories in distinct VNs. Bilateral assessments were performed. The stimulated EMG signals were continuously recorded. Any change in amplitude or latency was documented for each displacement. The amplitude and latency waveforms were separately displayed. The upper-limit threshold for latency (+ 10%) and lower-limit threshold for amplitude (-50%) were defined as separate alarm line dimensions^19,20^. Apart from the APS dislocation tests, a “positive VN stress test” was performed separately to find out the traction force needed to cause LOS of VN. The acoustic and optical signals would alert the surgeon when a preset threshold has been crossed, or when the electrode was displaced. In order to minimize the potential bias, all tests were conducted without any manipulation or traction on the trachea. The surgical field was kept dry with a swab to avoid any artifacts and\or signal shunting, and\or dispersion.

### Definitions and outcome evaluation

The following parameters were measured: tensile force of the APS detachment, EMG profiles before and during probe displacement, and the number of APS dislocations. A “positive VN stress test” was defined as an amplitude that decreases by > 50% and a latency that increases by > 10%. The strain parameters were measured in Newton (N), which was defined as the maximum force measured by the electronic force gauge during each detachment. Loss of signal (LOS) was defined according to the following criteria: initial EMG satisfactory (> 500 μV); no EMG response to stimulation at 1–2 mA or low response < 100 μV to stimulation at 1–2 mA; the troubleshooting algorithm was systematically applied^20^.

### Statistical analysis

A chart review was performed to obtain a detailed history of each identified case of APS displacement. All data were collected using an electronic Microsoft Office Access Database (Microsoft Corp, Redmond, Wash). Unless otherwise stated, all data were expressed as median and range. The statistical analysis was performed by Mann–Whitney test using SPSS 20.0 for Windows (SPSS Inc., Chicago-Ill, USA). The level of significance was set at *P* ≤ 0.05.

### Ethical approval

The Institutional Ethical Board Co mmittee approved the study (No. #2016 034 144). All experiments performed on the piglet were in accord ance to institutional guidelines, and compl ies with national and international regulations fo r animal experiments.

## Results

There were no adverse effects during the continuous monitoring. Furthermore, no cardiovascular sequelae were noted. In the present protocol study, the monitoring duration was 22 min on the right side and 25 min on the left side. The size of the VNs was 2.5 mm on the right side and 2.7 mm on the left side. The tests were repeated for > 50 times per side. The C-IONM electrode positioning was successfully performed without any adverse events, such as decreased amplitude or increased latency. The baseline parameters were as follows: bilateral amplitude > 1000 μV; right amplitude = 1950 µV; left amplitude = 2220 µV.

### Tensile strength for APS detachment

The 3-mm and 2-mm APS had a mean tensile strength of 20.6 ± 10 N (range, 14.6–24.4 N) and 11.25 ± 8 N (range, 8.4–15.6 N), respectively. The detachment of the electrode was easier to perform for the 2-mm accessories (*P* = 0.002). There was no difference between the bilateral VNs.

### EMG profiles before and during displacement

No electrode detachment exhibited LOS. The mean amplitude before and during electrode displacement was 1835 ± 102 μV and 1795 ± 169 μV, respectively (*P* = 0.45). The mean percentage of amplitude decrease on the EMG was 6.9 ± 2.5%, while the mean percentage of latency increase was 1.9 ± 1.5%. There was no statistical difference in amplitude decrease or latency increase between the left and right VNs (*P* = 0.5), or between the 2-mm and 3-mm APS (*P* = 0.45).

### Multiple APS dislocations

**Figure 3:**
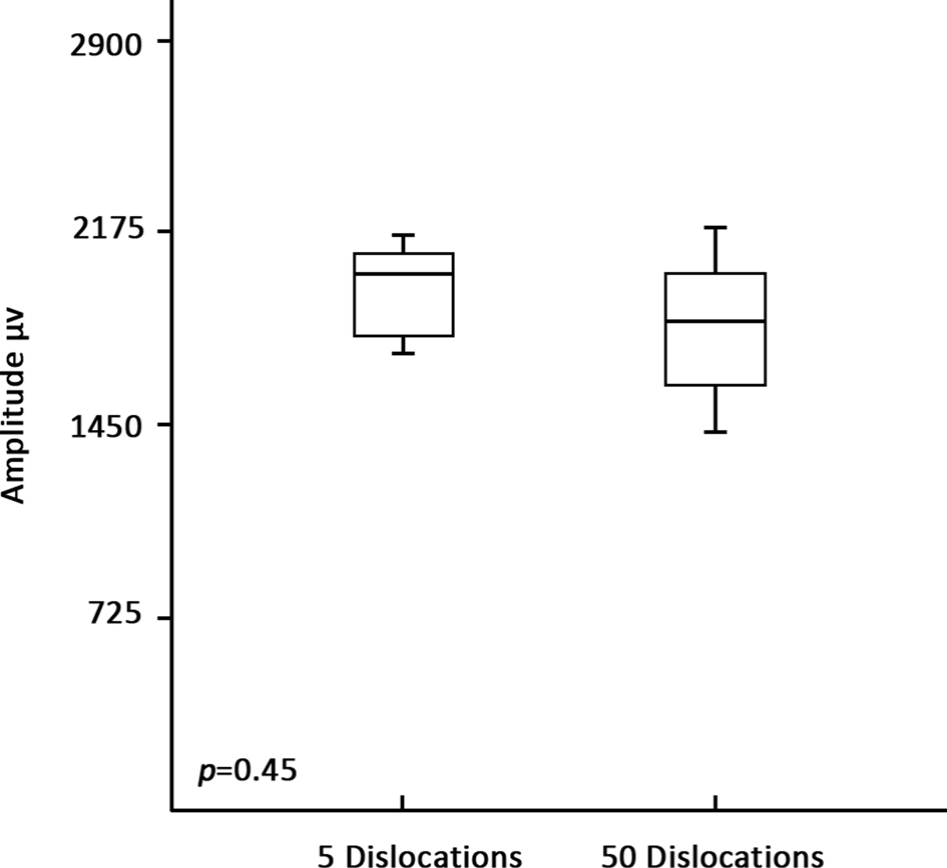
Amplitude profiles after multiple APS dislocations.

The amplitude profiles of multiple electrode dislocations are presented in Fig. 3. No significant amplitude reduction or LOS observed after 5 times and > 50 times of probe dislocations.

### VN stress test

No previous report has mentioned any traction force that causes LOS of VN. And our study failed to identify the force required for a 50% decrease in amplitude and 10% increase in latency, regardless of the multiple attempts per side, and the use of different sizes of APS. The amount of traction force that causes LOS of VN is far more than the force of an APS electrode detachment can generate. Due to the intrinsic characteristics, geometry and design of the electrode, the electrode signals never reached the alarm threshold.

## Discussion

The present experimental study provides an opportunity to examine the tensile effects of C-IONM probe migration on VN function, mimicking the continuous monitoring during thyroidectomy procedures. In an effort to accurately measure tensile strength for probe detachment, the investigators meticulously developed a translational piglet model. The study design included a proximal positioned recording APS, which provided a juncture to record changes after every APS displacement (Figs. 1 and 2).

The present study aimed to measure (a) the tensile force for APS detachment, (b) the EMG profiles before and during APS displacement, and (c) the EMG profiles after multiple APS dislocations.

The present results revealed that a 3-mm APS electrode requires a larger force to detach from VN than a 2-mm APS electrode. The mean percentage of EMG amplitude decrease and latency increase was 6.9% and 1.9%, respectively. These changes may be attributed to the artifacts or transient stress on the VN, since recovery was noted on the EMG. Unexpectedly, the present study revealed that multiple electrode dislocations did not alter the EMG profiles (Fig. 3).

The C-IONM electrode owns some adaptability. The actual size of the 2-mm electrode ranges within 2–3 mm, while that of the 3-mm electrode ranges within 3–4 mm^2,20^. However, the present study failed to identify the force required for a 50% decrease in amplitude and 10% increase in latency, regardless of the multiple attempts per side, and the use of different sizes of APS. Due to the intrinsic characteristics, geometry and design of the electrode, the electrode signals never reached the alarm threshold.

These present findings exhibit important significance for the qualitative and quantitative control of C-IONM probe positioning in clinical practice. In addition, the investigators attempted to reproduce the actual situation in clinical practice when using C-IONM in open, endoscopic, or robotic operations, namely, electrode dislocations due to the hindrance of retractors, gauzes, forceps, endoscopes and wires in the operative field. Surgeons have frequently reported that monitoring devices are usually interfered by surgical equipment, especially when a flexible wire is used for the APS.

In the future, the design and application of C-IONM electrodes should be simplified. Currently the most commonly used C-IONM electrode was analyzed, namely, the 2-mm and 3-mm APS. The investigators recommend surgeons to select the 2-mm APS due to its association with less tensile strength^2,3,19,20^. It is noteworthy that the mismatch between the VN size and electrode may result in poor stimulation or susceptibility to the electrode detachment^2,3^.

The main limitation of the present study was that no microscopic evaluation of the VN was performed, and that no comparisons were conducted among the different designs of C-IONM electrodes. That is, there may be an APS electrode that is easier to detach. Nevertheless, this could not be considered a shortcoming of the present use of APS, because the EMG data revealed that even after over 50 detachments, this was harmless to the VN. The present findings also confirm that APS is a safe tool, since there is relatively low risk for VN over-traction.

## Data Availability

All relevant data are presented in the manuscript.
